# A Goal Intervention Improves Language Fluency: Evidence from Parkinson’s Disease and Healthy Aging

**DOI:** 10.3390/medicines8030015

**Published:** 2021-03-22

**Authors:** Gail A. Robinson, Lara Campbell, Amelia Ceslis

**Affiliations:** 1 Queensland Brain Institute, The University of Queensland, Brisbane 4072, Australia; 2 Neuropsychology Research Unit, School of Psychology, The University of Queensland, Brisbane 4072, Australia; lara.campbell@uqconnect.edu.au (L.C.); a.ceslis@uq.edu.au (A.C.)

**Keywords:** initiation, apathy, Parkinson’s disease, language generation, narrative speech, verbal fluency, goal-directed behavior, goal intervention, executive functions, rehabilitation

## Abstract

**Background**: Parkinson’s disease [PD] is associated with reduced motor and cognitive initiation, and decreased goal-directed behavior including language generation. The current study investigated a novel goal intervention for language generation impairments in PD patients. **Methods:** Twenty-one PD patients and 22 healthy controls, matched for gender, age, and education, completed a cognitive baseline and language generation tasks (complex scene descriptions and phonemic/semantic word fluency) with standard and adapted instructions, which implements a target ‘goal’. In addition, participants completed self-report questionnaires for apathy and mood. **Results:** PD patients performed more poorly on two of three language generation tasks. The goal intervention was effective in increasing both the PD patient and healthy control groups’ language generation. However, there was no differential benefit of increased goal specificity and difficulty for PD patients. As a group, PD patients reported higher levels of apathy and depression than healthy controls. Specifically, PD patients with executive apathy were more likely to have language generation impairments than PD patients without executive apathy and controls. Apathy subscales and goal benefit were unrelated. **Conclusions:** The goal intervention was effective for PD patients and older adults, suggesting that enhanced goal specificity and difficulty may benefit individuals with PD or those aging naturally.

## 1. Introduction

Parkinson’s disease (PD) is a movement disorder associated with cognitive and behavioral symptoms, in addition to the characteristic motor symptoms [1,2]. The symptoms include slowness of initiation (akinesia), thought (bradyphrenia), and movement (bradykinesia), along with the hallmark tremors and rigidity [2]. PD is marked by deposition of alpha-synuclein and Lewy bodies with associated loss of dopaminergic neurons in the substantia nigra pars compacta and degradation of the frontostriatal pathways [3,4,5]. Disruption to frontostriatal circuits has been implicated in cognitive impairment affecting executive functions and initiation of spoken language [6,7,8,9], and can result in behavioral disorders such as apathy [10,11,12]. The current study aimed to investigate the initiation of spoken language and apathy in patients with PD, and the effectiveness of a novel goal intervention for improving language generation.

### 1.1. Language Generation

The voluntary expression of thoughts and ideas are integral to generating language for social communication [13]. Language generation goes beyond the core language skills of repetition, naming, and comprehension and incorporates a new idea given the current context [13]. Models of spoken language production generally agree on three main stages: (i) conceptualization or generation of ideas; (ii) linguistic formulation; and (iii) articulation [14,15,16,17,18,19,20]. The earliest conceptualization stage involves the initiation and generation of ideas, prior to the translation of an idea into the components of language (linguistic formulation), which are subsequently produced as overt speech (articulation).

Reduced idea generation is evident in sparse spontaneous speech, and it can be observed following a disturbance to frontal regions or frontostriatal circuits [21]. In PD, expressive language deficits can be evident at each stage of spoken language production including linguistic formulation (e.g., lexical-semantic and morphosyntactic processing [22]). Difficulties at the conceptualization stage can be observed as reduced content (i.e., spontaneous speech rate, simplified grammar) when describing complex scenes, or when asked open-ended questions or to provide a narrative on a topic [22,23] (for detailed review see [13]). Spontaneous speech impairment in the context of well-preserved core language skills (e.g., repetition, naming, reading, and comprehension) is the hallmark of dynamic aphasia, an acquired language disorder [24]. Patients with dynamic aphasia have been reported in the context of parkinsonian disorders (e.g., PD [25], progressive supranuclear palsy [21,26,27]), and following basal ganglia stroke [28,29]. 

Language generation tasks also comprise a significant executive functioning component. For instance, word fluency tasks are widely used to assess executive functioning [30]. Executive functions like task setting and monitoring are both required for successful word fluency performance to prevent set-loss and repetition errors [20]. PD patients are typically impaired on tests of executive functioning [31] including both verbal and non-verbal fluency tasks (e.g., [9]). This domain general impairment raises the possibility that reduced output is related to a general impairment in goal-directed behavior, secondary to executive dysfunction. In this regard, deficits in language generation could be due to impaired goal directed behavior rather than a language-specific ‘aphasia’.

### 1.2. Apathy

Apathy has been defined as “the quantitative reduction of self-generated voluntary and purposeful [or goal-directed] behaviors” ([10] p. 916). It is a behavioral disorder that is characterized by a loss of motivation and drive [11,12], which severely impacts daily activities, quality of life, survival, and caregiver burden [32,33]. It is common across a wide range of neurological and psychiatric disorders [34] and it is the most common behavioral symptom in PD dementia, observed in up to 50% of PD patients [35,36]. Despite its’ prevalence and impact, there is no effective therapy [34]. Therefore, it is important to identify apathy early in disease progression and develop novel interventions to reduce it and its’ widespread impact.

One reason apathy interventions have been unsuccessful to date is that apathy has several distinct dimensions. This multidimensionality is evident in conceptualizations, apathy scales, and diagnostic criteria (e.g., 2018 [34]). For instance, based on neuroanatomical regions involved and their corresponding mechanisms, Stuss and colleagues [12] proposed three apathy dimensions (cognitive, behavioral, and emotional), which were slightly modified by Levy and Dubois ([10]; cognitive, auto-activation, and emotional-affective, respectively) and Radakovic and Abrahams ([37]: executive, initiation, and emotion, respectively). These three dimensions are operationalized in apathy scales, for example, the Apathy Evaluation Scale (AES; [11]) and the Dimensional Apathy Scale (DAS; [37]). The current study used the AES as our study commenced prior to the DAS being available; however, we adopted the recent apathy dimension names of Radakovic and Abrahams [37] because these better reflect the language generation and executive function areas relevant for our goal intervention (see Appendix A for a comparison of the AES items and apathy dimensions according to framework).

With respect to the key neuroanatomical region and function of each dimension, executive apathy is associated with the dorsolateral frontostriatal circuit [10], which is key for executive functions such as task setting and monitoring [38]. Initiation apathy is associated with medial prefrontal dysfunction [10], a region key for initiating and sustaining behavior (i.e., energization; [38]). In contrast to executive and initiation apathy, emotion apathy, which is associated with the orbitofrontal region that is key for behavioral and emotional self-regulation, is less likely to be related to language generation [38]. This is because damage to orbitofrontal regions does not typically impact performance on standard executive or cognitive tests including language generation tasks like word fluency [38]. In summary, all three apathy dimensions may occur in PD either in isolation or in combination [10,39]. However, based on these dimensions and associated neuroanatomical regions, we hypothesize that only executive and initiation apathy are expected to be associated with language generation impairment due to differential mechanisms.

Of relevance is a patient with dynamic aphasia that, following a left thalamic stroke, was unable to *spontaneously activate or initiate* lexical-semantic representations, which could be activated by external stimuli (e.g., picture naming; [40]). The authors likened their patient’s impairment to a verbal form of the motor phenomena observed in PD known as kinesia paradoxica, which is thought to be due to disruption of the frontal–basal ganglia–thalamic network. This bears a striking resemblance to initiation apathy, described as ‘mental emptiness’ due to an energization impairment or inability to initiate and sustain responding on tasks [10,38] and it is reminiscent of Luria’s [24] description of dynamic aphasia as ‘an emptiness in the head’ (p. 208). Language generation tasks used to identify dynamic aphasia such as complex scene description and word fluency tasks are highly sensitive to impairment in energization (e.g., [6]) and initiation apathy [38]. Just as Levy and Dubois [10] proposed that reduced goal directed behavior in auto-activation (initiation) apathy can be reversed by external stimuli or prompting of actions, Luria and Tsvetkova [41] noted that external cues can help patients overcome dynamic aphasia by reorganizing and restoring the phrase. The effectiveness of prompts to overcome severely reduced spontaneous speech has been confirmed with dynamic aphasic patients [21,29]. Thus, there appears to be a close relationship between initiation (auto-activation) apathy and language generation impairments. Given that dynamic aphasia and initiation apathy have been noted to occur following basal ganglia lesions [10], it follows that initiation apathy may be associated with language generation impairments in PD patients [25].

### 1.3. Goal-Directed Behavior

Goal-directed behavior achieves an individual’s intention by purposeful action [42]. Goals may be immediate and physical such as quenching thirst, or long-term and abstract such as graduating from university [43]. Goals can originate from internal or external sources [44], with internally-generated rather than externally driven behaviors typically affected in disorders characterized by reduced goal-directed behavior such as that observed in PD. Two key factors affect goal-directed behavior in terms of setting a goal and performing a task; namely, *specificity* and *difficulty* [45]. First, specific and difficult goals direct attention toward goal-relevant stimuli (presumably via top-down attentional control). Second, specific, difficult goals have an energizing influence that increases effort and persistence on tasks. Finally, specific goals assist people to use task-relevant strategies and knowledge, and in terms of goal difficulty, the highest level of effort occurs when tasks are moderately difficult, that is neither too easy nor too hard [45]. These factors have been successfully implemented in neurorehabilitation contexts [46,47], as part of the S.M.A.R.T. (Specific Measurable Achievable Realistic Timed) goal framework [48]. To operationalize specific and difficult goals, the idea is to provide a goal that is defined with an external reference point, rather than simply ‘do your best’, and to set this at an attainable or realistic level [45]. For example, acquired brain injury patients performed faster when set an individual goal of 20% higher than their own baseline performance on a simple choice reaction time task [46]. In the current study, goal specificity and difficulty are manipulated in language generation tasks by modifying the task instructions (i.e., ‘goal’).

### 1.4. Current Study

In this study, we developed a novel behavioral intervention to target language generation, which is implicit in the initiation apathy dimension. This has been described as a lack of initiative [34,37,49] or diminished *initiation* of behaviors [11] such as starting conversations and initiation of goal-directed thoughts and actions [10]. Thus, initiation apathy is evident in language generation and manifests as diminished goal-directed activity in the form of reduced initiation and maintenance of conversation [34,50]. Within frontal lobe frameworks such as that described by Stuss [38], executive functions that organize and monitor behavior are integral, which suggests that executive apathy may manifest in a similar fashion.

The specific aims were to investigate: (1) Language generation performance of PD patients, compared to healthy controls, on spontaneous speech and word fluency tasks; (2) Whether a novel intervention of providing a goal improves language generation task performance; and (3) The presence of apathy in PD patients and whether apathy subtype moderates differences in language generation task performance or goal benefit. It is hypothesized that PD patients’ will be significantly reduced at baseline on language generation tasks and that a *specific* and *difficult* goal will enhance task performance. Furthermore, that all types of apathy will be present in the PD group. Regarding language generation, deficits are expected to occur in either initiation or executive apathy due to difficulty generating ideas per se, associated with an energization deficit [38], or difficulty selecting or sequencing messages [24], associated with executive dysfunction [38], respectively.

## 2. Materials and Methods

### 2.1. Participants

Participants included 21 PD patients who had received a diagnosis of PD according to the U.K. Brain Bank criteria [51] (see Table 1). The PD group was compared to 22 healthy controls without a neurological disorder. Both groups were recruited from the Queensland Parkinson’s Project and matched for gender, age, and education (all *p* > 0.05). All participants were right-handed (except one ambidextrous), as indicated by the Edinburgh Handedness Inventory [52]. PD patients were recruited from the database if they did not have documented cognitive deficits (a score of <24 on the Mini Mental State Examination) or a diagnosis of dementia (i.e., neurocognitive disorder). The Montreal Cognitive Assessment (MoCA) [53]) screening measure was given to both groups as an indicator of global cognition. As a group, PD patients scored lower than healthy controls on the MoCA, *t*(42) = −2.60, *p* = 0.014; however, individuals from both groups (five PD and three healthy controls) scored lower than 26/30, the screening cutoff for cognitive deficits in PD [54]. Clinical measures included disease duration, levodopa dose, the Unified Parkinson’s Disease Rating Scale (UPDRS; [55]) and the Hoehn and Yahr Scale [56]. The study was approved by the Royal Brisbane and Women’s Hospital (HREC/11/QRBWH/125; approved 28/06/2011) and the University of Queensland (#2011000187; approved 13/12/2011) human research ethics committees. Written informed consent was given by all participants.

### 2.2. Cognitive Tests and Self-Report Questionnaires

A series of cognitive tests were administered to characterize participants and ensure PD patients and healthy controls were comparable so that any benefit of the goal intervention was not attributable to other factors. Cognitive tests included measures of estimated premorbid intellectual functioning (National Adult Reading Test–2^nd^ edition (NART); [57]), fluid intelligence (Raven’s Advanced Progressive Matrices (APM); [58]), visual perception (Incomplete Letters test; [59]), sustained and selective auditory attention (Elevator Counting and Elevator Counting with Distraction; [60]), auditory-verbal working memory (digit span subtest; [61]), episodic verbal memory (Logical Memory subtest; [62]), language (sentence repetition; [63]; Object and Action Naming Test [64]; word comprehension–Synonym Judgment Test; [65]) and executive functions (Trail Making Test [66,67]; Tower Test [67]; Rule Shift Cards [68]; Hayling Sentence Completion and Brixton Spatial Anticipation Tests [69]; Balloon Analogue Risk Task (BART; [70]). 

To assess apathy, the apathy evaluation scale (AES; [11] was used. The AES is a well-validated self-rating scale that assesses cognitive (or executive), behavioral (or initiation), and emotional dimensions of apathy. Participants are asked to reflect on the past month and rate each of the 18 items as “very true”, “somewhat true”, “slightly true”, or “not at all true”. Each answer is scored from one to four with the sub scales and total score tallied. The Hospital Anxiety and Depression Scale (HADS; [71]) was used as a self-report measure of mood symptoms. The Behavioral Inhibition Scale/Behavioral Activation Scale (BIS/BAS) with four subscales (BAS Drive, BAS Reward Responsiveness, BAS Fun Seeking, BIS; [72]) was used to assess behavioral activation (or impulsiveness) and inhibition (or avoidance) to external (environmental) stimuli. PD patients have shown increased subjective BAS Reward Responsiveness, relative to healthy controls [73], and PD patients with elevated levels of apathy have reported lower pursuit of desired goals (BAS Drive) compared to PD patients without apathy [74].

### 2.3. Language Generation Tasks and Goal Intervention

#### 2.3.1. Complex Scene Description

Participants performed a baseline and experimental version of a complex scene description task in which they were asked to describe a pictorial scene as a measure of spontaneous connected speech. There were two scenes: (1) Cookie Theft Scene [75]; and (2) Beach Scene [76]. Participants were presented with each scene and given one minute to describe the contents. 

In the baseline (control) condition (Cookie Theft Scene), participants were given 1 min to *“Describe what’s going on in this picture”*. In the experimental (goal) condition, participants were presented with the Beach Scene and given 1 min to *“Speak continuously for one minute about this picture”.* This goal was designed to be more specific than “do your best” in the baseline condition. For both conditions, no specific prompts were given but the examiner asked ‘Anything else’ once, if there was a lengthy pause. Speech samples were transcribed for scoring purposes, with quantity calculated (words/minute) [21,27].

#### 2.3.2. Word Fluency

Participants performed three baseline and three experimental *Goal* trials of phonemic fluency, and two of each trial for semantic fluency. Participants were given a letter of the alphabet or a category and asked to orally generate as many different words as possible in 1 min, without including the names of people or places, or repeating the same word with different endings [77,78,79]. The total number correct minus the total errors produced (repetitions and rule-breaks) was averaged across the three phonemic and two semantic trials to yield a fluency score for each condition. 

Baseline Condition: In the baseline (control) condition, participants were instructed to “*Say as many words as you can that begin with the letter (F, A, S)*” for phonemic fluency and *“Name as many (Animals, Fruit & Vegetables) as you can”* for semantic fluency. 

Experimental *Goal* Condition: In the goal condition, participants were set an individual goal based upon their baseline performance. Their average performance across the three (phonemic) or two (semantic) baseline trials was computed, and their *goal was set as 20% higher than their baseline performance*. For example, if a participant generated 10 words for *F,* 9 words for *A,* and 11 words for *S*, their average baseline phonemic fluency performance was 10, and their goal was set at 12. They were then instructed to: *“Name 12 words beginning with the letter (B, M, W)”* for phonemic fluency. The letters FAS and BMW were chosen based on their equivalence in terms of rate of production in words per minute as well as error rates [80,81]. For semantic fluency, participants were instructed to *“Name 20% more than their average performance at baseline (Food in Trolley, Items of Clothing) in one minute”.* Correlations between different semantic categories were moderately high (0.66–0.71; [67]).

### 2.4. Statistical Analyses

A series of independent *t*-tests and Chi-square tests were used to compare the PD patient and healthy control groups on demographics, baseline cognitive tests, and self-report questionnaires. When the assumption of homogeneity of variance was not met, the Satterthwaite [82] method was used to adjust the error term and degrees of freedom. For the language generation measures, a series of 2 × 2 mixed model analyses of variance (ANOVAs) were used to assess performance on the complex scene description and word fluency tasks, with Group (PD vs. control) as the between and Goal (Goal vs. No Goal) as the within groups variables.

A case series approach was used to investigate the prevalence of clinical symptoms for three apathy dimensions for PD patients. Criteria for each apathy dimension was based upon descriptions by Levy and Dubois [10], although we adopted the recent Radakovic and Abrahams [50] apathy dimension names, as summarized in Table 2. Each PD patient was classified using these criteria. Statistically significant impairment on two or more tests or AES subscales was selected as the basis for the classifying executive and emotion apathy. This classification is stringent given that the recently revised apathy diagnostic criteria [34] only requires one form of evidence for each dimension for at least two of the three dimensions. Thus, statistically significant impairment on two or more of the criteria provides a more reliable and robust indication of genuine impairment in a dimension. The modified t-test was used for comparison of each PD patient to the control group [83,84] for each measure to ascertain impairment (e.g., on executive function tests used to classify executive apathy).

#### 2.4.1. Apathy and Language Generation

The relationship between apathy and language generation was explored at the group level using a series of bivariate correlations between the participants’ baseline language generation task performance (complex scene description, phonemic fluency, semantic fluency) and the apathy dimension criteria. The case series was then used to investigate the relative prevalence of language generation deficits in PD patients with different apathy subtypes. Chi-square test of independence could not be used due to the “expected” cell values being less than five, which violates the assumptions of chi-square [85,86]. Thus, Fisher’s exact test [85] was used to evaluate the frequency of deficits on each of the language generation tasks for PD participants with and without each of the apathy subtypes.

#### 2.4.2. Goal Intervention

The usefulness of the goal intervention was evaluated at the group level by calculating the “goal benefit” for each participant’s performance on the complex scene description, phonemic fluency, and semantic fluency tasks. Goal benefit is essentially a difference score, where the initial score in the No Goal condition is subtracted from the Goal condition score on each task. Thus, a positive goal benefit or difference score indicates improvement in the Goal condition, and a negative goal benefit score indicates poorer performance in the Goal condition, compared to the baseline. Goal benefit was then correlated with each of the criteria for the apathy subtypes described above. The case series was then resumed to investigate the pattern of goal benefit across PD patients with different apathy subtypes. Again, due to small sample sizes, Fisher’s exact test [86] was used to evaluate the frequency of goal benefit for participants with and without each apathy subtype on the three language generation tasks.

## 3. Results

### 3.1. Cognitive Tests and Self-Report Questionnaires

PD patients and healthy controls performed equivalently on measures of estimated pre-morbid intelligence, working memory, speed and attention, verbal episodic memory (recognition), language, and select tests of executive function (all comparisons *p* > 0.05) (see Table 3 for all cognitive tests). In contrast, PD patients performed worse than the controls on tests of non-verbal fluid intelligence (APM), *t*(39) = −2.76, *p* = 0.009, verbal episodic memory (immediate and delayed recall), *t*(39) = −2.42, *p* = 0.020 and *t*(39) = −2.79, *p* = 0.008, respectively, and tests of executive function that measure flexibility (Trail Making Test B), *t*(42) = 1.55, *p* = 0.023, inhibition (Card Shift Rule 2), *t*(37) = −2.56, *p* = 0.015, and nonverbal reasoning (Brixton Errors), *t*(32) = 2.21, *p* = 0.036. Although the PD and control groups differed on the visual perception test, *t*(39) = −3.04, *p* = 0.006, all participants performed close to ceiling and no PD patient performed lower than the 5^th^ percentile clinical cut-off score of 16 [59].

On self-report measures, relative to the control group, the PD group reported a significantly higher level of apathy, *t*(35) = 2.22, *p* = 0.033, and depression, *t*(38) = 3.82, *p* < 0.001, but not anxiety, *t*(35), = 1.52, *p* > 0.05, (see Table 4). At the individual level, four PD patients scored significantly higher than the control group for apathy, no PD patient scored above the clinical cut-off score of 11 for depression [71] and one PD patient scored above the clinical cut-off of 11 for anxiety [69]. The PD and control groups did not differ on any aspect of the Behavioral Activation and Inhibition measure including inhibition, drive, fun seeking, and reward responsiveness (all comparisons *p* > 0.05).

### 3.2. Spoken Language Tasks and Goal Intervention

The mean number of words produced on the complex scene description, phonemic fluency, and semantic word fluency tasks are presented in Table 5.

#### 3.2.1. Complex Scene Description

**Figure 1:**
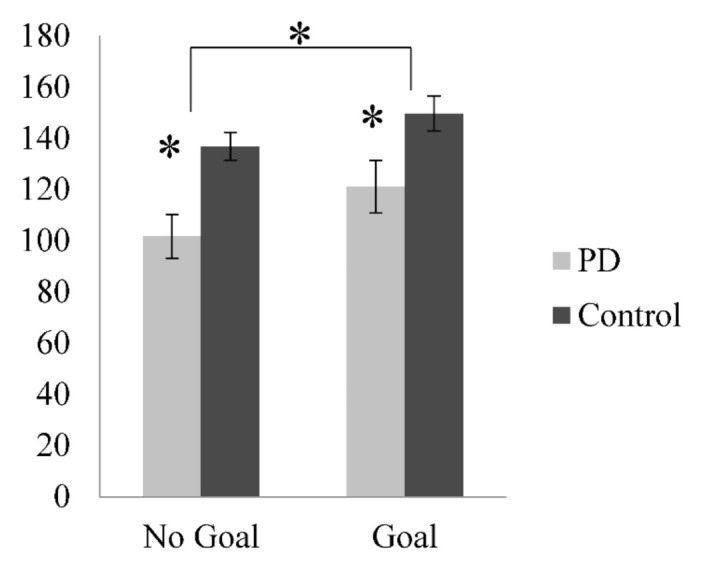
Speech rate (wpm) for Parkinson’s disease (PD) patients and healthy controls on the complex scene description task according to Goal condition; * *p* < 0.05.

Overall, there was a significant main effect of Group as the PD patients produced a significantly lower number of words in 1 min when describing the scenes compared to the healthy controls, *F*(1, 35) = 8.34, *p* = 0.007, η_p_^2^ = 192 (see Figure 1). In addition, there was a significant main effect of Goal as speech rate (words per minute [wpm]) was higher when given a more specific goal than with the standard baseline instructions, *F*(1, 35) = 7.46, *p* = 0.010, η_p_^2^ = 0.176. There was no interaction between Group and Goal, *F*(1, 35) = 0.24, *p* = 0.582, η_p_^2^ = 0.007.

#### 3.2.2. Word Fluency

**Figure 2:**
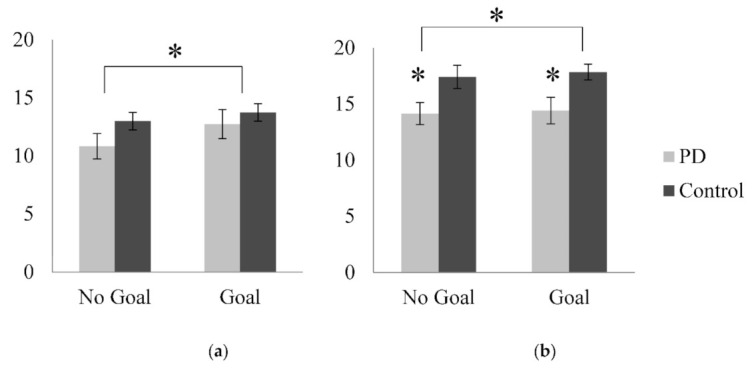
Mean number of words produced in one minute (wpm) by PD patients and healthy controls for (**a**) phonemic fluency and (**b**) semantic fluency according to the Goal condition; * *p* < 0.05.

For phonemic fluency, there was no significant main effect of Group in terms of average number of words produced in one minute, *F*(1, 37) = 1.19, *p* = 0.282, η_p_^2^ = 0.031. However, as shown in Figure 2a, there was a significant main effect of Goal, such that overall participants produced more words when given a specific goal than with standard baseline instructions, *F*(1, 37) = 11.91, *p* = 0.001, η_p_^2^ = 0.243. The interaction between Group and Goal was not significant, *F*(1, 37) = 2.30, *p* = 0.138, η_p_^2^ = 0.059.

For semantic fluency, there was a significant main effect of Group, *F*(1, 36) = 7.37, *p* = 0.010, η_p_^2^ = 0.170, as shown in Figure 2b. The PD patient group produced a significantly lower number of words per minute than the healthy controls. There was no significant main effect of Goal, *F*(1, 36) = 0.49, *p* = 0.488, η_p_^2^ = 0.013, and no significant interaction between Group and Goal, *F*(1, 36) = 0.05, *p* = 0.439, η_p_^2^ = 0.000.

### 3.3. Apathy Dimensions and Language Generation

In terms of apathy dimensions on the AES, the initiation (auto-activation) apathy subscale (seven items) was reliable in both samples (PD α = 0.80; Control α = 0.89). The 11-item emotion apathy subscale was also found to be highly reliable in both samples (PD α = 0.82; Control α = 0.82). As detailed above in Table 2, the executive (cognitive) apathy classification was derived from impairment on ≥2/5 tests of executive function. Of the PD patients, 33% were classified as having at least one subtype of apathy and 67% were classified without any apathy subtype. Of the 33% with apathy, the executive subtype was most prevalent (19%), followed by initiation apathy (14.3%) and then emotion apathy (9.5%).

The correlations between PD patients’ total apathy and depression scores and their baseline performance on each of the three language generation tasks were not significant (all *p* > 0.05). In terms of apathy dimensions, there were no significant correlations between any apathy subtype and the PD group’s speech rate on the complex scene description task. In contrast, for word fluency, there were significant positive correlations between phonemic fluency and digit span backwards, *r*(16) = 0.63, *p* = 0.004. Both fluency tasks significantly correlated positively with Card Shift Rule 2, *r*(17) = 0.63, *p* < 0.004 (phonemic)] and *r*(16) = 0.56, *p* = 0.012 (semantic) and negatively with the Trail Making B Test, *r*(18) = −0.53, *p* = 0.017 (phonemic), and *r*(18) = −0.72, *p* < 0.001 (semantic). No other correlations were significant including those between mood symptoms (i.e., depression and anxiety) and each of the three language generation tasks (all *p* > 0.05).

At the individual level, for the complex scene description and semantic fluency tasks, Fisher’s exact tests revealed there was a significantly higher frequency of impairment in PD patients with executive apathy compared to PD patients without executive apathy, *p* = 0.014 and *p* = 0.007, respectively (see Table 6). There were no significant effects for initiation or emotion apathy. For phonemic fluency, as no PD patient showed a significant deficit, there were no differential effects for the apathy subtypes.

### 3.4. Apathy Dimension and Goal Intervention

For the PD participants’ “goal benefit” score on the three language generation tasks, there were no significant correlations with overall apathy score or depression (all *p* > 0.05). For apathy subtype criteria, the only significant negative correlation was between semantic fluency goal benefit and digit span backwards, *r*(15) = −0.62, *p* = 0.007. The remaining correlations were not significant. 

At the individual level, for the complex scene description task, although it appeared to trend toward greater goal benefit within the “no apathy” group of PD patients, Fisher’s exact test yielded *p* = 0.268, thus goal benefit was unrelated to apathy status (see Table 7). For phonemic fluency, Fisher’s exact test yielded *p* = 1.000, thus goal benefit was also unrelated to apathy status for that task. Finally, for semantic fluency, Fisher’s exact test again yielded *p* = 1.000. In conclusion, there was no significant difference in the frequency of goal benefit between participants with and without apathy.

## 4. Discussion

This study presents a novel behavioral intervention designed to target reduced language fluency. Thus, the primary aim was to first investigate spontaneous language generation in PD patients and healthy controls and, second, ascertain whether a ‘goal’ intervention could improve language generation. Furthermore, we investigated the prevalence of apathy and its’ subtypes in PD patients, and whether this modulated either language generation per se or the capacity to benefit from the goal intervention. Our findings showed that the goal intervention was effective in increasing both the PD patient and healthy control groups’ language generation, despite no differential benefit of increased goal specificity and difficulty for PD patients. In addition, PD patients with executive apathy were more likely to have language generation impairments than PD patients without executive apathy and controls, although there was no relationship between apathy and goal benefit.

### 4.1. Language Generation

The PD patient group performed worse and produced a lower speech rate or number of words than healthy controls on the complex scene description and semantic fluency tasks, consistent with previous research (e.g., [25,49]). The absence of a phonemic fluency deficit for the PD patient group was not anticipated and is not in line with previous studies (e.g., [9,87]) or a meta-analysis that revealed both phonemic and semantic fluency deficits, albeit greater for semantic than phonemic fluency [88]. The latter meta-analysis concluded that greater semantic than phonemic deficits in PD were attributable to the additional semantic component that requires activation of semantic networks as well as executive functions. This may partly explain our significant language generation impairment for the two tasks with the greatest semantic component. With respect to phonemic fluency, our PD group produced an average of 10.9 words for the baseline task, which is of a comparable magnitude to other studies [9] despite not being significantly lower than our control groups’ mean of 12.9 words. Notably, the latter is somewhat lower than other studies (e.g., 15.3 [9]), which may at least partially account for the absence of a PD group deficit. Regarding semantic fluency, the PD group produced 14.1 words in the baseline condition, which was significantly lower than 17.4 words for the controls and falls in the low average range according to normative data [79], representing a mild impairment.

For the baseline complex scene description task, the PD groups’ speech rate was ~101 words per minute (wpm), which was significantly lower than the control group’s ~137wpm. In the context of well-preserved core language skills (repetition, naming, comprehension, reading), this represents a ‘subclinical’ form of dynamic aphasia as has recently been identified in other disorders (e.g., corpus callosal dysgenesis [89], amyotrophic lateral sclerosis [90]), even though the speech rate is not as reduced as that in the severe form of dynamic aphasia (e.g., ~21wpm [25]). Notably, patients with markedly reduced language generation and dynamic aphasia have been reported following disruption to frontostriatal circuits in parkinsonian disorders like PD [25] and PSP [21,26,27]), and after stroke to the basal ganglia [28,29]. As our PD group was selected because they were not globally impaired or showing signs of mild cognitive impairment (MCI) or dementia [91], the language generation impairments we document may represent the mild range of severity and earliest stage of degeneration. Early detection of cognitive deficits is critical to identify targets for intervention to enhance or maintain function in healthy or pathological aging (e.g., [92]).

Executive functions are integral for language generation with the interface between these receiving increasing attention (e.g., [13]). This is particularly since Alexander [93] explicitly discussed spontaneous speech as a complex goal-directed behavior implemented by three specific executive functions (energization, task-setting, and monitoring), which map onto the three key mechanisms identified as underlying dynamic aphasia. These key processes enable the speaker to initiate and sustain their attention on the intended focus (energization), decide what ideas are relevant to the focus (task-setting), and check whether the ideas produced are consistent with the focus (monitoring). These processes are also key for word fluency tasks that are widely used to assess executive functioning [30]. Specifically, energization, task-setting, and monitoring are key in ensuring sustained response initiation and to prevent set-loss (or rule-break) and repetition errors [38].

A further consideration is whether the language generation impairment is attributable to other factors. As our PD group was unimpaired on motor and verbal initiation speeded tasks (e.g., Trails Making MS/A, Hayling), this reduction is unlikely to be due to generalized psychomotor slowing that may have resulted in slow articulation or production. In addition, while the PD group self-reported a higher level of depression than the control group, this was at a sub-clinical level and did not correlate to baseline language generation task performance. This suggests that there is a genuine, albeit mild, language generation impairment on tasks with a high semantic component.

### 4.2. Goal Intervention and Language Generation

Providing a more *specific* and *difficult* goal resulted in enhanced performance for two language generation tasks (complex scene description and phonemic fluency). The notion to increase goal specificity and difficulty to improve performance on a task stems from goal setting theory [45]. Given the recognized role of executive functions in goal-directed behavior [43], and executive difficulties in PD [31], it is unsurprising that modifying goal factors was effective. However, there was an overall goal benefit across all participants that was not specific to PD patients. Although surprising, it is understandable in the context of healthy aging and the decline in key executive functions crucial for language generation (e.g., initiation, selection, inhibition, strategy [94,95,96]), and because of the loss of prefrontal neural structures and connections (e.g., [97,98]). Critically, this is the first study to demonstrate increased language output given a goal intervention. Moreover, it provides preliminary evidence that improving goal specificity and/or difficulty of the goal set (in the form of task instructions) can have a significant impact on task performance. The fact that participants showed a goal benefit in both the complex picture description and the phonemic fluency tasks provides support that it was a genuine goal benefit and not an artifact of the stimuli used. 

First, regarding the lack of goal benefit for the semantic fluency task, this may be due to the nature of the specific stimuli chosen. For the baseline condition, the categories of ‘animals’ and ‘fruit-vegetables’ were chosen and the categories of ‘food in trolley’ and ‘items of clothing’ were chosen for the goal condition. Despite research showing moderately high correlations between different semantic categories [67], this may not be true for our categories. For instance, while each category appears to comprise multiple subcategories with many exemplars (e.g., animals–farm, zoo, domestic, African, Australian) that lend themselves to the implementation of a strategy, ‘items of clothing’ may not contain as many items within subcategories as the other three. If this is the case, goal manipulation will not be as effective due to the limited scope for an increase between the baseline and goal conditions. In addition, although considerable care was put into matching the stimuli in both conditions to reduce the likelihood of stimuli artifacts, it is not possible to rule out a goal benefit due to practice effects as multiple trails were given. It is as equally likely that fatigue effects may have impacted performance, for instance, no goal benefit on the semantic fluency task.

From a theoretical executive functioning perspective, there are two possible explanations as to why *specific* (and/or) *difficult* goals facilitate performance on tasks. According to Stuss [38], the two core executive functions are task-setting and monitoring. Setting a more specific goal facilitates task-setting by better defining the criteria for success, making it easier to encode and translate the task instructions into an action plan. Specific goals are also likely to enable faster and less effortful monitoring of ongoing task performance. One way to determine which of these executive functions is responsible for the facilitatory effect of enhancing goal specificity and difficulty is to compare the performance of patients with focal left and right frontal lesions (e.g., similar methodology to [30]). This is because task setting difficulties are associated with left-sided frontal lesions, while monitoring is associated with right-sided frontal lesions [38]. It remains possible and indeed likely that both task setting and monitoring processes are facilitated by enhancing goal specificity.

One question that remains unresolved is whether the quantitative increase in words per minute on the complex scene description task under the goal condition represented a qualitative increase in meaningful and novel utterances. It is possible that the instruction to “speak continuously for one minute” led participants to repeat ideas or add words to sentences without generating new ideas. This would require detailed qualitative analysis of the content to examine novel ideas [21] and meaningfulness (e.g., coherence and cohesion [99]). This will be an important next step toward understanding the impact of goal factors on language generation.

### 4.3. Apathy Dimensions and Language Generation

Apathy was present in one third of the PD patients, which is comparable to prevalence rates in previous studies (e.g., 40% [100]). Our PD group also comprised each of the apathy subtypes, with executive apathy the most common followed by initiation and then emotion apathy. This is consistent with the suggestion by Levy and Dubois [10] that the most prevalent PD apathy presentation occurs due to dysfunction of the dorsolateral frontostriatal circuit, rather than the orbitofrontal or ventromedial circuits, and that this is associated with executive apathy.

As anticipated, there was a higher number of PD patients impaired on language generation tasks with executive or initiation apathy, compared to emotion apathy. Although it is logical to anticipate that participants with executive apathy would benefit most from the goal intervention due to the core deficit being in planning and organizing future goals (see Appendix B), we did not find evidence of a differential goal benefit within apathy dimensions. Additionally, we found a goal benefit for older adults that highlights the importance of task instructions for performance on cognitive testing. We note that the standard instructions for word fluency set a ‘do your best’ goal, which has consistently been shown to lead to poorer performance than specific, difficult goals [45].

One question that arises is about the specific mechanism for the goal benefit. The goal was effective even though we found differences within the PD patient group for some cognitive baselines and for the presence or absence of apathy. While our PD and control groups were well matched on demographic variables and most baseline cognitive tests including working memory, language, processing speed and attention, the PD group performed more poorly on tests of verbal memory (free recall), nonverbal reasoning, flexibility, and inhibition. These impairments are consistent with the pattern observed in PD as these cognitive tests are largely ‘executive’ functions, associated with frontal regions and frontostriatal dysfunction in PD (e.g., [5,31]). Notably, these mild and select baseline cognitive deficits did not preclude the PD group from benefitting from the goal. We highlight that our PD group was ‘high-functioning’ and largely intact or at the mild end of the range of PD severity for cognitive deficits. It would be of interest to investigate whether PD patients with MCI or dementia benefit from a goal to the same degree as our PD group, if at all.

## 5. Conclusions

This is the first study to demonstrate that a specific and difficult goal increases language fluency. The novel goal intervention was effective for PD patients and older adults, which suggests that enhanced goal specificity and difficulty may benefit healthy and pathological aging. Our goal intervention is a simple behavioral technique, with potential applications within a wide range of neurorehabilitation and community settings. For instance, modifying task instructions to facilitate improved performance can be integrated as a general principle within neurorehabilitation. Clinicians are welcome to use the wording for the goal interventions presented in our paper, for example, asking patients to “keep talking” or “talk to me for one minute about X”. Furthermore, increasing language generation is critical for social interaction and the communication of thoughts and needs in daily life, which then significantly impacts an individuals’ quality of life. 

## Figures and Tables

**Table 1:** Demographics for Parkinson’s disease (PD) Patients and Healthy Controls (means and standard deviations).

	PD Patients *n* = 21		Healthy Controls *n* = 22	
	*M*	*SD*	*M*	*SD*
Sex (Male:Female)	14:7	-	10:12	-
Age (Years)	70.24	5.98	66.81	8.68
Education (Years)	13.36	3.29	13.84	3.53
MoCA (/30)	25.43	3.82	27.87	2.10
Disease Severity				
Years since Diagnosis	8.38	5.26	-	-
UPDRS	64.56	18.54	-	-
Hoehn & Yahr	2.69	0.87	-	-

*Note.* MoCA = Montreal Cognitive Assessment; UPDRS = Unified Parkinson’s Disease Rating Scale.

**Table 2:** Apathy dimensions: Method of classification based on Levy and Dubois [10].

Executive Apathy (Cognitive)	Initiation Apathy (Auto-Activation)	Emotion Apathy (Emotional-Affective)
Significantly impaired on ≥2/5 executive function tests (Trail Making B Test, Digit Span Backwards, Brixton, Tower, Card Shift 2)	Significantly elevated above controls on AES Initiation Apathy subscale (see Appendix A)	Significantly elevated above controls on AES Emotion Apathy subscale (see Appendix A) BART BIS/BAS Reward Responsiveness

*Note.* AES = Apathy Evaluation Scale; BART = Balloon Analogue Risk Task; BIS/BAS = Behavioral Inhibition Scale/Behavioral Activation Scale.

**Table 3:** Cognitive tests for PD patients and healthy controls.

	PD Patients *n* = 21		Healthy Controls *n* = 22	
	*M*	*SD*	*M*	*SD*
Intellectual Functioning					
NART Estimated FSIQ	111.15	10.52	113.00	9.62
Raven’s APM (/12)	6.05 *	3.53	8.65	2.37
Visual Perception					
Incomplete Letters (/20)	19.14 *	1.20	19.95	0.22
Working Memory					
Digit Span Total (/30)	17.85	4.28	18.95	4.83
Digit Span Forward (/16)	10.70	2.62	11.45	2.80
Digit Span Backward (/14)	7.15	2.32	7.50	2.26
Speed and Attention					
Trail Making Test Motor Speed (s)	34.05	13.08	28.30	10.22
Trail Making Test A (s)	50.44	46.07	34.41	17.67
Elevator Counting (/7)	6.87	0.52	7.00	0.00
Elevator Count + Distraction (/10)	7.56	3.18	7.73	2.55
Memory					
Verbal Immediate (/75)	35.79 *	8.52	44.23	12.98
Verbal Delayed (/50)	20.21 *	6.84	27.82	10.02
Verbal Recognition (/30)	24.31	3.61	25.35	3.54
Language					
Sentence Repetition (/10)	9.67	0.66	9.91	0.29
Object Naming (/79)	77.950	2.12	78.65	0.59
Synonyms Total (/60)	57.17	2.68	58.75	1.73
Executive Functioning					
Trail Making Test B (s)	119.45 *	84.91	72.11	30.13
BART (average adjusted pumps)	21.55	9.20	24.62	8.36
Tower Total Achievement Score (/30)	16.60	6.52	17.59	3.18
Card Shift Rule 1 (/20)	19.81	0.87	20.00	0.00
Card Shift Rule 2 (/20)	17.90 *	2.15	19.32	1.11
Brixton SS (/10)	4.00	2.66	5.44	1.86
Brixton Errors (total)	23.61 *	10.11	17.50	5.65
Hayling SS (/10)	4.50	1.99	5.35	1.37

*Note.* NART = National Adult Reading Test; APM = Advanced Progressive Matrices; BART = Balloon Analogue Risk Task; SS = Scaled Score. * *p* < 0.05.

**Table 4:** Self-report measures for PD patients and healthy controls.

	PD Patients *n* = 21		Healthy Controls *n* = 22	
	*M*	*SD*	*M*	*SD*
Apathy Evaluation Scale (/72)	31.70 *	8.14	25.94	7.52
HADS Anxiety (/21)	5.05	2.91	3.65	2.91
HADS Depression (/21)	4.63 **	2.90	1.75	1.71
Behavioral Inhibition Scale (/28)	18.25	2.32	20.31	3.89
BAS: Drive (/16)	10.75	2.59	9.25	2.18
BAS: Fun Seeking (/16)	11.16	2.03	10.38	2.22
BAS: Reward Response (/20)	15.50	2.92	16.12	2.52

*Note.* HADS = Hospital Anxiety and Depression Scale; BAS = Behavioral Activation Scale; * *p* < 0.05; ** *p* < 0.01.

**Table 5:** Language generation tasks: Number of words produced in 1 min for the baseline and goal Intervention for PD patients and controls.

	PD Patients *n* = 21		Healthy Controls *n* = 22	
	*M*	*SD*	*M*	*SD*
Picture Elicited Narrative Task				
Baseline (Cookie)	104.58	40.75	136.83	24.81
Goal (Beach)	122.95	43.18	149.67	29.35
Phonemic Fluency				
Baseline (FAS)	10.93	4.72	12.98	3.43
Goal (BMW)	12.92	5.38	13.75	3.26
Semantic Fluency				
Baseline (F&V, Animals)	14.05	4.21	17.42	4.50
Goal (Food, Clothing)	14.39	4.87	17.84	3.08

*Note.* F&V = Fruit and Vegetables.

**Table 6:** Language generation deficit and apathy subtype: Number of impaired PD participants.

	No Apathy	Executive Apathy	Initiation Apathy	Emotion Apathy
Complex Scene Description				
Deficit: No Deficit	3:10	4:0	3:1	1:2
*% with Deficit*	*23%*	100% *	*75%*	*50%*
Phonemic Fluency				
Deficit: No Deficit	0:13	0:4	0:4	0:4
*% with Deficit*	*0%*	*0%*	*0%*	*0%*
Semantic Fluency				
Deficit: No Deficit	2:11	4:0	3:1	1:6
*% with Deficit*	*15%*	100% *	*75%*	*14%*

Note. * *p* < 0.05

**Table 7:** Goal intervention benefit and apathy subtype: Number of PD participants that benefitted.

	Controls	PD No Apathy	PD Executive Apathy	PD Initiation Apathy	PD Emotion Apathy
Complex Scene Description					
Goal Benefit: No Goal Benefit	12:6	9:2	2:1	1:2	1:1
*% with Benefit*	*67%*	*82%*	*67%*	*33%*	*50%*
Phonemic Fluency					
Goal Benefit: No Goal Benefit	11:8	10:2	3:1	2:1	2:0
*% with Benefit*	*58%*	*83%*	*75%*	*67%*	*100%*
Semantic Fluency					
Goal Benefit: No Goal Benefit	12:7	5:6	2:2	1:2	2:0
*% with Benefit*	*63%*	*45%*	*50%*	*33%*	*100%*

## Data Availability

The conditions of our ethics approval do not permit public archiving of the data supporting the conclusions of the study. Readers seeking access to this data should contact the corresponding author (G.A.R.). Access will be granted to named individuals in accordance with ethical procedures governing with reuse of sensitive data. Specifically, requestors must complete a formal data sharing agreement and have local ethics approval to obtain the data.

## Abbreviations

PD = Parkinson’s disease; AES = Apathy Evaluation Scale; DAS = Dimensional Apathy Scale; MoCA = Montreal Cognitive Assessment; UPDRS = Unified Parkinson’s Disease Rating Scale; BART = Balloon Analogue Risk Task; BIS/BAS = Behavioral. Inhibition Scale/Behavioral Activation Scale; NART = National Adult Reading Test; APM = Advanced Progressive Matrices; BART = Balloon Analogue Risk Task; SS = Scaled Score.

## Appendix A

**Table A1:** Comparison of apathy evaluation scale items according to the original subscale [11], Levy and Dubois apathy subtypes [10], and the current apathy dimensions, based on Radakovich and Abrahams [37].

Apathy Evaluation Scale Item	Marin et al.	Levy & Dubois	Current
1. I am interested in things	Cognitive	Emotional-Affective	Emotion
2. I get things done during the day	Behavioral	Auto-Activation	Initiation
3. Getting things started on my own is important to me	Cognitive	Auto-Activation	Initiation
4. I am interested in having new experiences	Cognitive	Emotional-Affective	Emotion
5. I am interested in learning new things	Cognitive	Emotional-Affective	Emotion
6. I put little effort into anything *	Behavioral	Auto-Activation	Initiation
7. I approach life with intensity	Emotional	Emotional-Affective	Emotion
8. Seeing a job through is important to me	Cognitive	Auto-Activation	Initiation
9. I spend time doing things that interest me	Behavioral	Emotional-Affective	Emotion
10. Someone has to tell me what to do each day *	Behavioral	Auto-Activation	Initiation
11. I am less concerned about my problems than I should be *	Cognitive	Emotional-Affective	Emotion
12. I have friends	Behavioral	Emotional-Affective	Emotion
13. Getting together with friends is important to me	Cognitive	Emotional-Affective	Emotion
14. When something good happens, I get excited	Emotional	Emotional-Affective	Emotion
15. I have an accurate understanding of my problems	Other	Emotional-Affective	Emotion
16. Getting things done during the day is important to me	Cognitive	Auto-Activation	Initiation
17. I have initiative	Other	Auto-Activation	Initiation
18. I have motivation	Other	Emotional-Affective	Emotion

*Note.* Items are scored on a 4-point Likert scale: Not at all true (1), Slightly true (2), Somewhat true (3), Very true (4); * denotes item that is not reverse scored. All other items are reverse scored.

## Appendix B

**Table A2:** Apathy subtypes, corresponding neural and functional impairments, and suggested measures.

Lesion or Dysfunction	Clinical Signs	Measures
EXECUTIVE APATHY		
Dorsolateral Prefrontal Cortex Basal ganglia (cognitive) Anterior Thalamic Nuclei	↓ planning & organizing goals: *(Poor s* elf-generation, switching, spontaneous retrieval, working memory, set maintaining)	↓ performance on cognitive tests (Tower, Word Fluency, Trail Making B, Brixton, Logical Memory II Digit Span Backwards, Card Shifting)
INITIATION APATHY		
Medial Prefrontal Cortex (Superior Frontal Gyrus and Anterior Cingulate Cortex) Basal ganglia (cognitive/limbic)	Short-lived emotional responses ↓ spontaneous activation of mental set/emotional response ↓ self-generation of thoughts ↑ behaviors in response to external solicitation	→ AES3, AES6 → AES8, AES16, AES17, AES2 → ↑ performance with external cues → AES10
EMOTION APATHY		
Orbitomedial Prefrontal Cortex Basal ganglia (limbic)	Emotional blunting Loss of interest in activities Decreased reward sensitivity Decreased social involvement	→ AES7, AES14, AES11, AES15 → AES1, AES4, AES5, AES9, AES18 → BAS Reward Responsiveness Scale → Gambling and reversal tasks → AES12, AES13

*Note.* AES = Apathy Evaluation Scale.
